# Validation of the Mongolian version of the SF-36v2 questionnaire for health status assessment of Mongolian adults

**DOI:** 10.1186/s40064-016-2204-7

**Published:** 2016-05-12

**Authors:** Motoyuki Nakao, Keiko Yamauchi, Yoko Ishihara, Bandi Solongo, Dashtseren Ichinnorov, Raoul Breugelmans

**Affiliations:** Department of Public Health, School of Medicine, Kurume University, 67 Asahimachi, Kurume, Fukuoka 830-0011 Japan; Department of Respiratory Medicine, School of Medicine, Mongolian National University of Medical Sciences, Ulaanbaatar, Mongolia; Department of Medical Education, Tokyo Medical University, Tokyo, 160-8402 Japan

**Keywords:** SF-36, COOP/WONCA charts, Reliability, Validity, Mongolia, Japan, Pulmonary function, Respiratory symptom, Air pollution

## Abstract

**Background:**

Ulaanbaatar, Mongolia, is one of the world’s worst air-polluted cities, but effects of this air pollution on the population health status have not yet been evaluated. Therefore, we developed a Mongolian version of the SF-36v2 questionnaire to investigate the health status of Mongolian population.

**Methods:**

Health checkups were conducted in Ulaanbaatar and the health status was measured using a Mongolian translated version of the SF-36v2 questionnaire. The reliability and validity of the Mongolian SF-36v2 questionnaire, and the relationship between health status and respiratory condition were examined.

**Results:**

Factor analysis of the Mongolian SF-36v2 questionnaire showed that the “Role-physical” and “Role-emotional” were classified into a single subscale. The “Mental health” and “Vitality” were each divided into two subscales. Cronbach’s alpha and intraclass correlation coefficient (ICC) for reproducibility were >0.7, except for “General health perceptions” (Cronbach’s alpha and ICC < 0.7), “Social functioning” (Cronbach’s alpha < 0.7), and “Vitality” (ICC < 0.7). The SF-36v2 subscales and the corresponding items of the COOP/WONCA charts were correlated, and subjects with respiratory symptoms showed lower SF-36v2 scores compared to normal subjects, suggesting external validity. Subjects with respiratory symptoms showed significantly lower scores for the majority of the SF-36v2 subscales than those with normal lung function. In subjects with combined ventilatory impairment, “Physical functioning”, “Role-physical”, “Bodily pain”, and “Vitality” scores were significantly lower than those with normal lung function.

**Conclusions:**

The Mongolian version of the SF-36v2 questionnaire provides substantial reliability and validity, and is useful for evaluating the health status of Mongolian adults with ventilatory impairment. Health status measured by SF-36v2 was significantly aggravated by combined ventilatory impairment when compared with normal lung function.

**Electronic supplementary material:**

The online version of this article (doi:10.1186/s40064-016-2204-7) contains supplementary material, which is available to authorized users.

## Background

Ulaanbaatar, the capital of Mongolia, is one of the world’s worst air-polluted cities and there have been growing concerns about air pollution from anthropogenic sources (WHO 2014). A remarkable increase in coal and biomass fuel consumption during the winter months is the major cause of air pollution in Ulaanbaatar, in addition to its topographic characteristics as a basin (Amarsaikhan et al. 2014). Increased coal consumption during winter is primarily due to an increased household use of coal-fired stoves or boilers. This increase in household coal consumption is contributed by a population influx from rural areas to Ulaanbaatar, leading to the development of ger districts, which are characterized by ger clusters comprising traditional Mongolian nomadic tents with poor infrastructure. During winter, coal or biomass-fired stoves used in each ger is a major cause of frequent smog in Ulaanbaatar. Four coal-fired electric power stations in Ulaanbaatar are also a significant source of air pollution. However, the health effects of this air pollution on the Ulaanbaatar populace have not yet been fully evaluated. The lack of appropriate questionnaires and medical supplies such as spirometers are contributing factors to this dearth of research in Mongolia. Although Enkhjargal et al. reported that the air pollution was associated with respiratory diseases especially among children, there has been no report on the effect of air pollution on the respiratory health of adult or elderly subjects (Enkhjargal et al. 2010). Sonomjamts et al. and Viinanen et al. have independently reported on the prevalence of asthma and allergic rhinitis among Mongolian subjects (Sonomjamts et al. 2014; Viinanen et al. 2005). However, there has been no report on the association between respiratory diseases and the health status of Mongolian adults.

Short-Form 36 Health Survey (SF-36v2) is a suitable tool for assessing health-related quality of life and has been used in many physical health conditions and healthcare settings (Aaronson et al. 1998; Garratt et al. 1993). It measures health status as the extent to which physical health impacts on functional ability and perceived well-being in mental, social and physical aspects of life. The SF-36v2 utilizes a scoring system based on eight functional health and well-being categories: physical functioning (PF), role limitations due to physical health problems (RP), bodily pain (BP), general health perceptions (GH), Vitality (VT), social functioning (SF), role limitations due to emotional problems (RE) and mental health (MH). The SF-36v2 has been translated into many languages and is widely used as a multilingual questionnaire in consideration of participant cultural background, such as language and customs (Li et al. 2003; Hoopman et al. 2006; Guermazi et al. 2012; Mbada et al. 2015). However, there is currently no Mongolian version of the SF-36v2.

In this study, we translated the original English version of the SF-36v2, which is widely used globally for health status measurement, into a Mongolian version. We then conducted a study to evaluate the usefulness of this Mongolian SF-36v2 questionnaire for health status assessment of Mongolian adults living in Ulaanbaatar.

## Methods

### Translation of the English SF-36v2 and COOP/WONCA charts into Mongolian

Translation of the English SF-36v2 (Table 1) into Mongolian was carried out with permission of QualityMetric Inc. (New York, NY, USA), the copyright holder of the SF-36v2, in accordance with the written directives (McHorney et al. 1993). Briefly, the English SF-36v2 was translated into Mongolian and after back translation, the expressions and lexicon were assessed by native Mongolian and English speakers. The translation was revised several times to ensure both conceptual equivalence with the original English version and ease of understanding by the target language population. The Mongolian SF-36v2 was then pilot tested through cognitive debriefing interviews of regular Mongolian citizens, after which QualityMetric staff, in collaboration with the translation team, reviewed the contents thoroughly and obtained final approval of the Mongolian version after minor adjustments. The English version of the COOP/WONCA charts was translated into Mongolian with permission of van Weel, the author of the original version (van Weel 1993). The translation process was similar to that of the SF-36v2. Permission to use the Japanese version of SF-36v2 was also obtained from Quality Metric. For the Japanese version of the COOP/WONCA charts, permission was obtained from the Japan Primary Care Association.

**Table 1:** Questions of SF-36v2 English version

Question	
Q1	In general, would you say your health is: (1 = Excellent to 5 = Poor)
Q2	Compared to 1 year ago, how would you rate your health in general now? (1 = Much better now than 1 year ago to 5 = Much worse now than 1 year ago)
Q3	The following questions are about activities you might do during a typical day. Does your health now limit you in these activities? If so, how much? (1 = Yes, limited a lot to 3 = No, not limited at all)
	(a) Vigorous activities, such as running, lifting heavy objects, participating in strenuous sports
	(b) Moderate activities, such as moving a table, pushing a vacuum cleaner, bowling, or playing golf
	(c) Lifting or carrying groceries
	(d) Climbing several flights of stairs
	(e) Climbing one flight of stairs
	(f) Bending, kneeling, or stooping
	(g) Walking more than a mile
	(h) Walking several hundred yards
	(i) Walking one hundred yards
	(j) Bathing or dressing yourself
Q4	During the past 4 weeks, how much of the time have you had any of the following problems with your work or other regular daily activities as a result of your physical health? (1 = All of the time to 5 = None of the time)
	(a) Cut down on the amount of time you spent on work or other activities
	(b) Accomplished less than you would like
	(c) Were limited in the kind of work or other activities
	(d) Had difficulty performing the work or other activities (for example, it took extra effort)
Q5	During the past 4 weeks, how much of the time have you had any of the following problems with your work or other regular daily activities as a result of any emotional problems (such as feeling depressed or anxious)? (1 = All of the time to 5 = None of the time)
	(a) Cut down on the amount of time you spent on work or other activities
	(b) Accomplished less than you would like
	(c) Did work or other activities less carefully than usual
Q6	During the past 4 weeks, to what extent has your physical health or emotional problems interfered with your normal social activities with family, friends, neighbors, or groups? (1 = Not at all to 5 = Extremely)
Q7	How much bodily pain have you had during the past 4 weeks? (1 = None to 6 = Very severe)
Q8	During the past 4 weeks, how much did pain interfere with your normal work (including both work outside the home and housework)? (1 = Not at all to 5 = Extremely)
Q9	These questions are about how you feel and how things have been with you during the past 4 weeks. For each question, please give the one answer that comes closest to the way you have been feeling. How much of the time during the past 4 weeks… (1 = All of the time to 5 = None of the time)
	(a) Did you feel full of life?
	(b) Have you been very nervous?
	(c) Have you felt so down in the dumps that nothing could cheer you up?
	(d) Have you felt calm and peaceful?
	(e) Did you have a lot of energy?
	(f) Have you felt downhearted and depressed?
	(f) Did you feel worn out?
	(h) Have you been happy?
	(i) Did you feel tired?
Q10	During the past 4 weeks, how much of the time has your physical health or emotional problems interfered with your social activities (like visiting friends, relatives, etc.)? (1 = All of the time to 5 = None of the time)
Q11	How TRUE or FALSE is each of the following statements for you? (1 = Definitely true to 5 = Definitely false)
	(a) I seem to get sick a little easier than other people
	(b) I am as healthy as anybody I know
	(c) I expect my health to get worse
	(d) My health is excellent

### Self-completed questionnaire and health survey

A self-completed questionnaire package was administered containing questions regarding age, sex, occupation, and respiratory symptoms, and the translated SF-36v2 and COOP/WONCA charts. At the time of questionnaire administration, height, body weight, blood pressure, and peripheral capillary oxygen saturation (SpO_2_) were measured, and a medical interview and auscultation by a physician were also conducted. A pulmonary function test was performed with the HI-105 spirometer (CHEST M.I., Inc., Tokyo, Japan) where the forced vital capacity (FVC) and forced expiratory volume in one second (FEV_1_) were measured and the FEV_1_/FVC ratio was calculated. Subjects were classified into four groups according to the guidelines of The Japanese Respiratory Society; normal, obstructive (FEV_1_/FVC ratio <70 %), restrictive (FVC < 80 % of predicted value), and combined ventilatory impairment (FEV_1_/FVC ratio <70 % and FVC < 80 % of predicted value) (Sasaki et al. 2001).

### Subjects

Health surveys were carried out at eight community clinics located all over Ulaanbaatar from 2012 to 2013. Participants were recruited by announcements advertising the health survey for male and female subjects aged 40–79 years. Administration of the self-completed questionnaire, body measurements, and medical examination including the pulmonary function test were performed in male and female community volunteers aged 40–79 years (Table 2).

**Table 2:** Demographic characteristics of participants

	Mongolian (n = 705)		
	Male	Female	Welch’s *t* test
[n (%)]	258 (37)	447 (63)	
Age [mean ± SD]	53.4 ± 10.6	54.0 ± 9.5	n.s.

			χ^2^-test
Age group [n (%)]			
40–49	107 (41)	156 (35)	n.s.
50–59	79 (31)	163 (36)	
60–69	44 (17)	96 (21)	
70–79	28 (11)	32 (7)	
Smoking status [(n (%)]			
Smoker	140 (54)	46 (10)	<0.0001
Ex-smoker	34 (13)	16 (4)	
Non-smoker	84 (33)	385 (86)	
Occupation [n (%)]			
White collar job	23 (9)	63 (14)	<0.0001
Blue collar job	131 (51)	145 (32)	
Not in employment	103 (40)	230 (51)	
Missing	1 (0)	9 (2)	

Seven hundred and thirty-seven self-reported questionnaires were collected, and 32 subjects were excluded from analysis due to an incomplete SF-36v2 or COOP/WONCA charts. A total of 705 (95.7 %) collected questionnaires were eligible for analysis.

Twenty-nine healthy volunteers were recruited for reproducibility assessment of the Mongolian SF-36v2. Those involved in this reproducibility assessment were tested using the Mongolian SF-36v2, and re-tested after 1 week using the same questionnaire.

Japanese subjects recruited in the Kumamoto prefecture were also investigated. Subjects who visited the healthcare center were recruited into our study. Administration of the self-completed questionnaire, body measurements, and medical examination were also performed in male and female participants aged 40–79 years, and 855 subjects were eligible for analysis. Demographic characteristics of Japanese participants are shown in Additional file 1: Table S1. Significant gender differences were found in the smoking status and occupation category for both countries.

### Data handling and statistical analyses

All data were handled by questionnaire ID, and managed as electronic data for the analysis. SF-36v2 scores were converted to subscale scores according to the Manual of the Japanese version of the SF-36v2 (Fukuhara and Suzukamo 2004). Each subscales was based on questions in the original version of the SF-36v2 as follows; PF, Q3 (a–j); RP, Q4 (a–d); BP, Q7 and Q8; GH, Q1 and Q11 (a–d); VT, Q9 (a, e, g, i); SF, Q6 and Q10; RE, Q5 (a–c); MH, Q9 (b, c, d, f, h). Statistical analyses including Welch’s *t* test for parametric analyses of continuous variables of two groups, Steel–Dwass test following Kruskal–Wallis test for non-parametric analysis of continuous variables, χ^2^-test for analyses of categorical data, factor analysis using varimax rotation, and calculations for Spearman’s correlation coefficient and intraclass correlation were carried out on the statistical software packages JMP version 11 (SAS Institute Inc., Cary, NC, USA) or SPSS ver. 21.0 (IBM Corp., Armonk, NY, USA). *P* values less than 0.05 were considered to be statistically significant.

### Ethical considerations

The present study was approved by the Clinical Ethical Review Board of Kurume University School of Medicine. Before investigation, participants were provided with explanations in person as to the purpose and method of the study, as well as information regarding the handling of the results. The study was carried out upon receipt of written consent.

## Results

### Construct validity, reliability and reproducibility

The English version of the SF-36v2 consists of eight subscales PF, RP, BP, GH, VT, SF, RE and MH. Factor analysis was carried out using the Mongolian version of the SF-36v2 to test the construct validity (Table 3). For the PF, BP, and GH subscales, the Mongolian SF-36v2 consisted of the same items as the English SF-36v2. The RP and RE subscales were consolidated into a single factor that is different from the English SF-36v2. The MH subscale was divided into two subscales, with one as the same factor as SF, and the other as the same factor as VT. Two of the questions comprising the VT subscale were classified as another independent factor. In the Japanese SF-36v2, the RP, GH, BP, and RE subscales consisted of the same items as the English version. Although the VT and MH factors were composed of two factors, the questions making up these subscales were not identical to the English SF-36v2. One of the factors of the VT/MH subscales included questions in the SF subscale. Two of the questions that constituted the PF subscale were classified as another independent factor (Additional file 2: Table S2). Internal consistency reliability was assessed by calculating Cronbach’s α (Table 4). Cronbach’s α calculated for eight subscales exceeded 0.7 (range 0.711–0.892) in six subscales except for VT (α = 0.544) and SF (α = 0.599). The reproducibility of the questionnaire was examined using the test–retest method, and the retest was carried out on the same subject after an interval of 1 week (Table 4). The eight subscales showed significantly high correlation (Spearman’s correlation coefficients 0.624–0.948) between the test and retest. Intraclass correlation coefficients (ICC) exceeded 0.7 (range 0800–0.943) in six subscales except for GH [0.600, 95 % confidence interval (CI) 0.303–0.792] and VT (0.692, 95 % CI 0.440–0.844).

**Table 3:** Factor analysis of the Mongolian SF-36v2

Mongolia (n = 705)	Factor 1	Factor 2	Factor 3	Factor 4	Factor 5	Factor 6	Factor 7	Factor 8
Q1	−0.230	−0.125	−0.189	*0.477*	0.107	0.272	−0.074	−0.034
Q2	−0.140	−0.099	−0.095	0.288	0.022	0.240	−0.068	−0.039
Q3								
(a)	*0.553*	0.220	0.042	−0.151	0.041	−0.074	0.056	−0.151
(b)	*0.670*	0.248	0.036	−0.116	0.029	−0.046	0.039	−0.162
(c)	*0.650*	0.224	0.094	−0.051	−0.018	−0.052	0.007	−0.125
(d)	*0.667*	0.141	0.042	−0.200	0.032	−0.097	0.017	0.013
(e)	*0.596*	0.177	0.090	−0.039	0.003	−0.083	0.047	0.151
(f)	*0.598*	0.109	0.089	−0.148	0.010	−0.109	0.083	0.112
(g)	*0.672*	0.167	0.086	−0.168	−0.022	−0.072	0.115	0.314
(h)	*0.608*	0.185	0.074	−0.148	−0.015	−0.095	0.068	0.536
(i)	*0.587*	0.189	0.121	−0.068	−0.009	−0.053	0.052	0.533
(j)	*0.516*	0.247	0.110	−0.113	−0.029	−0.024	0.046	0.212
Q4								
(a)	0.273	*0.685*	0.105	−0.202	−0.013	−0.115	0.045	0.030
(b)	0.212	*0.671*	0.129	−0.172	−0.009	−0.035	0.097	0.048
(c)	0.349	*0.713*	0.088	−0.123	−0.017	−0.086	0.073	0.081
(d)	0.331	*0.695*	0.155	−0.137	−0.035	−0.102	0.103	0.048
Q5								
(a)	0.171	*0.726*	0.259	−0.090	−0.051	−0.128	0.059	0.035
(b)	0.199	*0.694*	0.266	−0.123	−0.055	−0.065	0.104	0.052
(c)	0.243	*0.658*	0.297	−0.128	−0.092	−0.099	0.091	0.009
Q6	−0.086	−0.253	−*0.355*	0.177	0.151	0.260	−0.078	−0.008
Q7	−0.244	−0.214	−0.238	0.296	0.084	*0.746*	−0.109	−0.044
Q8	−0.254	−0.323	−0.305	0.271	0.148	*0.559*	−0.178	−0.030
Q9								
(a)	0.042	−0.035	−0.072	0.071	*0.675*	0.011	0.010	−0.010
(b)	0.106	0.229	*0.649*	−0.139	−0.093	−0.131	0.133	0.049
(c)	0.069	0.190	*0.703*	−0.075	−0.096	−0.096	0.095	0.043
(d)	0.032	−0.016	−0.175	0.052	*0.768*	0.016	−0.034	−0.047
(e)	−0.054	−0.100	0.073	0.204	*0.676*	0.072	−0.053	0.002
(f)	0.089	0.201	*0.717*	−0.068	−0.054	−0.030	0.122	0.012
(g)	0.127	0.239	0.374	−0.171	0.019	−0.148	*0.701*	0.061
(h)	0.044	0.005	−0.119	0.104	*0.760*	0.054	0.087	0.037
(i)	0.181	0.240	0.369	−0.231	0.046	−0.129	*0.667*	0.018
Q10	0.155	0.287	*0.385*	−0.188	−0.135	−0.136	0.143	0.004
Q11								
(a)	0.143	0.172	0.170	−*0.305*	−0.140	−0.034	0.142	0.047
(b)	−0.169	−0.138	−0.047	*0.601*	0.151	0.086	−0.010	−0.004
(c)	0.104	0.183	0.140	−*0.479*	−0.092	−0.084	0.103	0.085
(d)	−0.136	−0.106	−0.034	*0.831*	0.120	0.037	−0.054	0.029

The highest factor loadings in each observed variable were expressed as italic

**Table 4:** Internal consistency, test–retest reliability, and intraclass correlations of the Mongolian SF-36v2

SF-36v2 subscales	Internal consistency reliability (Cronbach’s α) (n = 705)	Test–retest reliability (n = 29)	
		Spearman’s correlation coefficient	Intraclass correlation coefficient (ICC) (95 % CI)
Physical functioning	0.892	0.948	0.943 (0.883–0.973)
Role limitations due to physical problems	0.876	0.838	0.829 (0.666–0.917)
Bodily pain	0.884	0.891	0.880 (0.760–0.943)
General health perceptions	0.743	0.624	0.600 (0.303–0.792)
Vitality	0.544	0.707	0.692 (0.440–0.844)
Social functioning	0.599	0.921	0.921 (0.839–0.963)
Role limitations due to emotional problems	0.862	0.820	0.800 (0.616–0.902)
Mental health	0.711	0.709	0.833 (0.670–0.920)

### External validity

Individual scores of the eight subscales of the SF-36v2 and the corresponding items of the COOP/WONCA charts were highly correlated (Table 5): the PF subscales of the SF-36v2 showed the highest correlation coefficient with “Pain”, the RP, VT, SF, and RE subscales corresponded to “Daily activities”, and the BP, GH, SF, and MH subscales corresponded to the “Pain”, “Overall health”, “Social activities”, and “Feelings” scales of the COOP/WONCA charts, respectively.

**Table 5:** Correlation of SF-36v2 subscale scores and the COOP/WONCA charts scores

SF-36v2	COOP/WONCA charts						
	Physical fitness	Feelings	Daily activities	Social activities	Overall health	Pain	Quality of life
Physical functioning	−0.219	−0.217	−0.340	−0.197	−0.306	−0.382	−0.167
Role limitations due to physical problems	−0.110	−0.308	−0.406	−0.282	−0.321	−0.388	−0.231
Bodily pain	−0.105	−0.359	−0.448	−0.317	−0.490	−0.699	−0.316
General health perceptions	−0.192	−0.350	−0.387	−0.258	−0.517	−0.476	−0.335
Vitality	−0.111	−0.321	−0.435	−0.309	−0.350	−0.397	−0.348
Social functioning	−0.051	−0.371	−0.408	−0.397	−0.324	−0.368	−0.320
Role limitations due to emotional problems	−0.094	−0.327	−0.430	−0.285	−0.308	−0.355	−0.292
Mental health	−0.091	−0.477	−0.412	−0.356	−0.261	−0.322	−0.435

The relationships between the SF-36v2 subscale score and the respiratory symptoms were analyzed (Table 6). Subjects who answered positive on question 1 (Q1), “Does the weather affect your cough?” showed significantly lower scores on six out of eight subscales except for RE and VT, compared to those who answered “No cough”. When compared with subjects without sputum production in the absence of a cold, subjects who answered positive on Q2, “Do you ever cough up sputum from your chest when you don’t have a cold?” showed significantly lower scores on all subscales. For Q3, “Do you usually cough up sputum from your chest first thing in the morning?”, subjects who answered in the affirmative showed significantly lower scores than subjects who answered in the negative in seven subscales except for MH. Subjects with frequent wheezing showed significantly lower scores in five subscales except for RP, RE, and VT compared to those who did not wheeze frequently. Subjects who answered positive on Q5, “Do you have or had any allergy?” showed significantly lower scores on seven subscales except for PF compared to subjects without any allergy.

**Table 6:** Effects of respiratory symptoms on the scores of SF-36v2 subscales

SF-36v2 subscales (n = 705)	Q1: Does the weather affect your cough?			Q2: Do you ever cough up sputum from your chest when you don’t have a cold?		Q3: Do you usually cough up sputum from your chest first thing in the morning?		Q4: How frequently do you wheeze?		Q5: Do you have or had any allergy?	
	Yes	No	No cough	Yes	No	Yes	No	Occasionally or more often	Never	Yes	No
PF	60.6 ± 24.7	65.0 ± 25.8	67.9 ± 27.1**	57.8 ± 25.0	66.3 ± 25.6****	56.2 ± 25.4	65.9 ± 25.1****	60.7 ± 24.8	64.8 ± 26.0*	60.8 ± 25.0	63.7 ± 25.8
RP	62.5 ± 24.9	68.3 ± 25.8	69.2 ± 28.7*	60.3 ± 25.3	68.8 ± 26.2****	57.9 ± 27.0	68.2 ± 25.2****	64.6 ± 25.3	65.8 ± 26.7	60.6 ± 26.6	66.3 ± 25.9*
RE	64.9 ± 25.4	69.2 ± 25.3	72.2 ± 27.8	62.3 ± 26.4	71.2 ± 25.3****	61.0 ± 27.8	70.0 ± 24.9****	66.8 ± 25.4	68.1 ± 26.5	62.8 ± 27.9	68.7 ± 25.5*
BP	55.2 ± 23.5	61.2 ± 26.2*	37.6 ± 27.0****	55.2 ± 24.2	62.2 ± 25.8***	53.0 ± 23.8	61.7 ± 25.5****	55.1 ± 22.7	62.2 ± 26.5***	51.6 ± 23.5	61.2 ± 25.4****
GH	48.4 ± 21.9	51.6 ± 21.6	57.5 ± 22.9****	46.3 ± 22.4	54.5 ± 21.6****	44.9 ± 23.2	53.6 ± 21.5****	47.9 ± 21.1	53.5 ± 22.6***	43.9 ± 20.7	53.0 ± 22.3****
VT	59.4 ± 18.9	61.7 ± 20.1	63.3 ± 18.9	57.6 ± 18.9	63.1 ± 19.1***	57.2 ± 20.0	62.3 ± 18.8***	59.5 ± 18.5	61.8 ± 19.5	55.3 ± 20.3	62.1 ± 18.7***
SF	72.5 ± 22.1	76.3 ± 24.2	78.4 ± 22.8	71.4 ± 23.7	77.0 ± 21.9***	71.8 ± 24.1	75.8 ± 22.3*	72.7 ± 22.3	76.1 ± 22.8*	69.8 ± 24.7	75.8 ± 22.2***
MH	68.1 ± 19.4	68.3 ± 21.1	72.2 ± 17.3**	66.5 ± 20.5	70.9 ± 18.4***	67.4 ± 20.5	69.8 ± 19.0	67.3 ± 19.0	70.3 ± 19.4*	64.8 ± 21.1	70.1 ± 18.9***

Data are presented as mean ± SD

SF-36v2 subscales: *PF* physical functioning, *RP* role limitations due to physical health problems, *RE* role limitations due to emotional problems, *BP* bodily pain, *GH* general health perceptions, *VT* vitality, *SF* social functioning, *MH* mental health

* P < 0.05; ** P < 0.01; *** P < 0.005; **** P < 0.0001 vs subjects who answered “yes” (“Occasionally or more” for Q4)

### Effects of ventilatory impairment and physiological parameters on SF-36v2 subscale scores

**Fig. 1:**
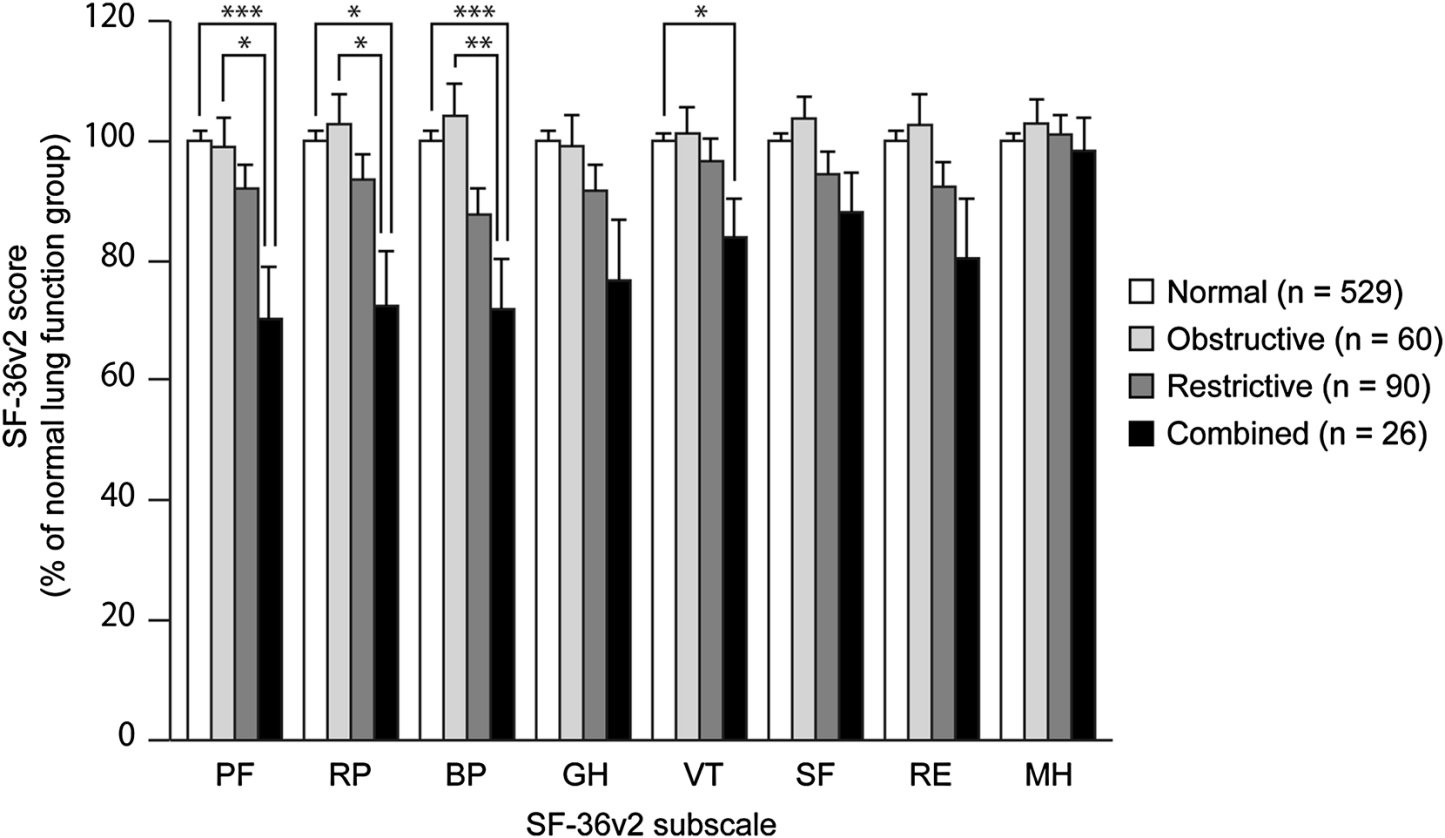
Effects of lung function on SF-36v2 subscales scores in Mongolian subjects. SF36v2 subscales; *PF* physical functioning, *RP* role limitations due to physical health problems, *BP* bodily pain, *GH* general health perceptions, *VT* vitality, *SF* social functioning, *RE* role limitations due to emotional problems; and *MH* mental health. Data are presented as mean percentage of the mean score of subjects with normal lung function ± standard error of the mean. Nonparametric multiple comparisons were carried out by Steel–Dwass test on each pair following Kruskal–Wallis test. *P < 0.05; **P < 0.01; ***P < 0.005 between each group

Pulmonary function tests in all subjects were performed by trained staff. The subjects were classified into four groups according to the results of the pulmonary function test: normal lung function, obstructive, restrictive, or combined ventilatory impairment. Figure 1 shows the effect of ventilatory impairment on the SF-36v2 subscale scores. When compared with the scores of subjects with normal lung function, the scores in the combined ventilatory impairment group were significantly lower for PF, RP, BP, and VT. Subjects with combined ventilatory impairment also showed significantly lower scores for PF, PR, and BP when compared with subjects with obstructive ventilatory impairment. There were no other significant differences in scores, although the scores tended to be lower in restrictive and combined ventilatory impairment.

Effects of physiological parameters such as body mass index (BMI), SpO_2_, FVC, FEV_1_, FEV_1_/FVC ratio, systolic and diastolic blood pressure on SF-36v2 subscale scores were assessed by gender (Table 7). Subjects were divided in two groups according to the median of each parameter. In males, PF score was lower in subjects with lower SpO_2_, FVC, and FEV_1_. Lower RP and MH scores correlated with lower FVC scores, and MH score was further lowered by lower BMI. Scores in RE, BP, GH, VT and SF subscales were not significantly affected by any physiological parameters. In females, PF scores were lower in those with higher BMI and blood pressure, and lower FVC and FEV_1_. Lower FVC and FEV_1_ also lowered BP scores, and MH scores were higher in those with lower SpO_2_. Lower RP and RP scores correlated with higher systolic blood pressure. GH, VT and SF subscales were not significantly affected by any physiological parameters.

**Table 7:** Effects of physiological parameters on the scores of SF-36v2 subscales

	SF-36v2 subscales^a^							
	PF	RP	RE	BP	GH	VT	SF	MH
*Male (n* = *258)*								
BMI^b^								
≥Median	65.8 ± 23.8	64.9 ± 27.1	69.6 ± 26.6	66.5 ± 25.3	58.0 ± 23.2	64.4 ± 18.4	80.6 ± 20.6	74.8 ± 19.0
<Median	68.8 ± 27.2	66.4 ± 27.1	68.0 ± 26.6	64.7 ± 27.5	54.5 ± 24.0	61.1 ± 20.5	76.9 ± 22.1	69.7 ± 19.3*
SpO_2_								
≥Median	71.3 ± 24.3	67.2 ± 26.6	70.5 ± 25.2	67.4 ± 27.6	56.9 ± 23.6	63.5 ± 19.1	80.3 ± 21.3	72.2 ± 18.9
<Median	62.0 ± 27.2**	63.7 ± 27.8	65.9 ± 28.3	62.5 ± 24.9	54.4 ± 23.9	60.8 ± 20.5	75.6 ± 21.8	71.0 ± 19.9
FVC								
≥Median	75.9 ± 21.1	69.3 ± 24.8	69.5 ± 26.0	67.5 ± 27.2	58.7 ± 23.9	62.0 ± 19.0	78.0 ± 21.6	68.3 ± 19.4
<Median	60.1 ± 27.5****	62.6 ± 28.7*	68.0 ± 27.1	63.6 ± 26.1	53.4 ± 23.3	62.7 ± 20.4	78.9 ± 21.6	74.8 ± 18.7**
FEV_1_								
≥Median	75.3 ± 21.6	69.2 ± 24.2	69.7 ± 25.2	66.7 ± 26.6	58.2 ± 24.2	63.0 ± 18.5	77.7 ± 21.2	69.8 ± 18.3
<Median	60.7 ± 27.4****	62.8 ± 29.2	67.8 ± 27.8	64.3 ± 26.7	53.9 ± 23.1	61.9 ± 20.8	79.1 ± 21.9	73.5 ± 20.0
FEV_1_/FVC								
≥Median	70.0 ± 24.6	66.2 ± 26.5	68.9 ± 24.3	65.7 ± 26.2	58.4 ± 23.5	63.5 ± 19.3	77.2 ± 21.5	69.5 ± 18.5
<Median	65.5 ± 26.8	65.5 ± 27.6	68.5 ± 28.5	65.2 ± 27.1	53.7 ± 23.7	61.5 ± 20.0	79.6 ± 21.6	73.8 ± 19.7
Systolic blood pressure								
≥Median	67.1 ± 26.0	65.7 ± 28.1	69.7 ± 26.6	67.3 ± 26.6	57.6 ± 23.2	61.7 ± 21.0	79.1 ± 22.3	71.3 ± 21.1
<Median	68.1 ± 25.8	65.9 ± 26.1	67.6 ± 26.6	63.6 ± 26.6	54.2 ± 24.1	63.1 ± 18.4	77.7 ± 20.9	72.2 ± 17.3
Diastolic blood pressure								
≥Median	68.9 ± 24.2	66.2 ± 27.4	69.0 ± 26.7	67.0 ± 26.5	56.3 ± 22.8	61.8 ± 20.3	78.2 ± 21.7	71.3 ± 20.7
<Median	66.3 ± 27.6	65.4 ± 26.9	68.4 ± 26.7	63.7 ± 26.8	55.5 ± 24.7	63.1 ± 19.1	78.7 ± 21.5	72.2 ± 17.6
*Female (n* = *447)*								
BMI^b^								
≥Median	56.8 ± 24.6	62.9 ± 26.6	64.9 ± 27.1	54.5 ± 23.8	46.8 ± 21.0	60.1 ± 19.5	72.2 ± 23.9	68.3 ± 19.5
<Median	64.4 ± 25.1***	67.2 ± 24.4	69.1 ± 24.4	57.2 ± 24.1	50.1 ± 21.1	59.5 ± 18.2	72.9 ± 22.6	66.7 ± 19.1
SpO_2_								
≥Median	61.0 ± 25.0	65.5 ± 24.8	65.6 ± 25.8	54.8 ± 24.8	48.5 ± 19.8	58.8 ± 19.3	71.6 ± 22.5	65.8 ± 18.8
<Median	60.0 ± 25.3	64.5 ± 26.6	68.7 ± 25.9	57.3 ± 22.8	48.4 ± 22.7	61.1 ± 18.2	73.8 ± 24.2	69.8 ± 19.7*
FVC								
≥Median	63.9 ± 24.0	66.4 ± 25.0	68.0 ± 25.0	59.1 ± 24.4	48.3 ± 20.6	60.6 ± 19.1	71.5 ± 22.5	66.4 ± 18.5
<Median	57.5 ± 25.8**	63.8 ± 26.1	66.0 ± 26.6	52.8 ± 23.1**	48.6 ± 21.5	59.1 ± 18.5	73.5 ± 22.9	68.5 ± 20.0
FEV1								
≥Median	63.5 ± 24.6	66.2 ± 26.1	67.6 ± 25.7	58.4 ± 23.9	49.5 ± 20.9	60.3 ± 19.2	71.7 ± 22.1	66.1 ± 19.4
<Median	57.9 ± 25.3*	64.0 ± 25.1	66.4 ± 26.0	53.5 ± 23.8*	47.5 ± 21.2	59.4 ± 18.5	73.3 ± 24.3	68.8 ± 19.2
FEV_1_/FVC								
≥Median	62.6 ± 25.5	66.8 ± 26.2	66.3 ± 25.6	57.6 ± 23.4	49.3 ± 20.4	59.8 ± 18.2	73.3 ± 22.9	66.6 ± 19.4
<Median	58.8 ± 24.6	63.5 ± 24.9	67.6 ± 26.1	54.3 ± 24.4	47.7 ± 21.7	59.8 ± 19.4	71.8 ± 23.6	68.3 ± 19.2
Systolic blood pressure								
≥Median	56.8 ± 26.0	62.4 ± 25.6	64.1 ± 25.9	54.1 ± 23.4	48.5 ± 21.3	59.3 ± 18.8	72.5 ± 23.7	67.7 ± 20.1
<Median	64.4 ± 23.6***	67.7 ± 25.3*	69.8 ± 25.6*	57.6 ± 24.4	48.4 ± 20.9	60.3 ± 18.9	72.6 ± 22.9	67.3 ± 18.5
Diastolic blood pressure								
≥Median	57.9 ± 25.2	63.5 ± 25.7	65.6 ± 26.3	55.0 ± 23.6	49.1 ± 20.6	59.9 ± 18.3	73.0 ± 23.6	68.6 ± 19.4
<Median	63.5 ± 24.7*	66.7 ± 25.4	68.5 ± 25.3	56.8 ± 24.3	47.7 ± 21.6	59.7 ± 19.4	72.1 ± 22.9	66.4 ± 19.2

* P < 0.05; ** P < 0.01; *** P < 0.005; **** P < 0.0001 versus ≥median

^a^SF-36v2 subscales: *PF* physical functioning, *RP* role limitations due to physical health problems, *RE* role limitations due to emotional problems, *BP* bodily pain, *GH* general health perceptions, *VT* vitality, *SF* social functioning, MH Mental health

^b^BMI body mass index (kg/m^2^)

## Discussion

The present study was carried out to clarify the factors affecting the health status of Mongolian subjects, particularly those with ventilatory impairment, due to growing concerns about air pollution from anthropogenic sources. As a tool for evaluating the health status of Mongolian subjects, the Mongolian version of the SF-36v2 showed generally satisfactory construct validity although the Mongolian subjects aged 40–79 years in this study did not distinguish between the RP and RE subscales. Additionally, the MH subscales were divided into the SF and VT subscales. Internal consistency reliability was confirmed by Cronbach’s α, and the reliability examined by the test–retest method showed significantly high correlations with ICCs that were of substantially high value to confirm the reproducibility. In addition, the external validity of the Mongolian SF-36v2 was confirmed through correlation with COOP/WONCA charts and questions regarding respiratory symptoms. These results suggest that this questionnaire is useful for evaluating differences in health status between Mongolian subjects with normal lung function and those with ventilatory impairment.

The SF-36v2 has been translated into many languages and is widely used as a multilingual questionnaire for the measurement of health status differences between normal subjects and patients with various diseases such as multiple sclerosis (Fernandez et al. 2011), rheumatoid arthritis (Matcham et al. 2014), schizophrenia (Papaioannou et al. 2011), chronic obstructive pulmonary disease (Prieto et al. 1997), cardiovascular disease (Jenkinson et al. 1997; Dempster and Donnelly 2001), and cancer (Mosconi et al. 2002). However, no Mongolian SF-36v2 has been developed to date. Therefore, we developed the Mongolian version of the SF-36v2 questionnaire in collaboration with seven language professionals with permission from QualityMetric over the course of more than 2 years. We investigated 737 Mongolian subjects, which equaled approximately 0.1 % of the Mongolian population aged 40 years or more, from autumn of 2012 to winter of 2013. Mongolians aged 40 years or more account for 26.6 % of the total Mongolian population (United Nations 2014, 2015).

The construct validity of the Mongolian version of the SF-36v2 was assessed by factor analysis. The result showed that the subscales regarding mental components, which consist of the VT, SF, RE and MH subscales, were not clearly divided as with the English version, in contrast to the subscales regarding physical components, which consist of the PF, RP, BP and GH, although the RP and RE subscales were consolidated in the Mongolian subjects. Nevertheless, the subscales of the physical component were independent of each other. Both the RP and RE subscales of the SF-36v2 were highly correlated with the “Daily activity” subscale of the COOP/WONCA charts. This result suggests that Mongolians recognized that limitations in daily activities were mainly due to physical health problems rather than emotional or mental problems. In their study assessing the construct validity of the SF36v2 covering patients with mental illness and healthy volunteers, McHorney et al. demonstrated that RP correlated with medical severity and psychometrics, and RE correlated with psychiatric disorder and psychiatric severity (McHorney et al. 1993). However, Ware et al. performed a principal component analysis of the SF36v2 in a study of ten countries in Europe and the USA, and demonstrated that RP and RE were race-independent for the physical component score and the mental component score, respectively (Ware et al. 1998). We previously reported that factor analysis of the SF-36v2 collected from 783 citizens of the general populace in Japan and China aged 50–79 years showed that the RP and RE subscales were clearly divided (Yamaguchi et al. 2013). These results suggest that recognition of physical and psychological roles in daily activities is independent of age, sex and race. With respect to occupation, approximately 40 % of Mongolian male and 51 % of female subjects were not in employment or retired in this study. Relatively high unemployment rates were also shown in our previous study where 32.6 % of Japanese male, 45.8 % of Japanese female, 20.2 % of Chinese male, and 39.1 % of Chinese female subjects were not in employment or retired (Yamaguchi et al. 2013). Therefore, the effect of occupational status on the recognition of the role in daily activity was suggested to be small. Japanese and Chinese have quite different lifestyles and worldviews when compared with Mongolians, probably because Japanese and Chinese are essentially settled agricultural people compared to Mongolians who comprise nomadic, hunter-gatherer tribes. This difference may be a reason why Mongolians consider physical and mental aspects of daily activities as a unified concept. Further investigation is required to clarify the Mongolian-specific concept of the physical-mental interface.

SF-36v2 was validated as a tool for evaluating health-related quality of life in patients with symptomatic chronic obstructive pulmonary disease (COPD), characterized by persistent airway obstruction caused by a mixture of small airway disease and parenchymal destruction (Mahler and Mackowiak 1995). We therefore analyzed the effects of each physiological parameter on SF-36v2 subscales in participants with ventilatory impairment. SF-36v2 subscale scores were analyzed by sex because these parameters differ between sexes (Table 7). Physiological parameters such as FVC, FEV_1_ and SpO_2_ mainly affected physical aspects of the health status such as PF and RP subscales in male subjects, and BMI, FVC, FEV1 and blood pressure mainly affected PF, RP and BP subscales in female subjects with the exception of several mental aspects such as RE and MH. GH, VT and SF subscales were not significantly affected by any physiological parameters measured in the present study. The most susceptible subscale of SF-36v2 was PF in both sexes although the effect varied according to sex. It has been reported that there is sex-difference of SF-36v2 scores (Hajian-Tilaki et al. 2016; Kitaoka et al. 2016; Prata et al. 2016). Nevertheless, the subscales which differed by sex were entirely different between these reports and the present study because of differences in subjects’ age, clinical background and ethnic group. Therefore, further investigation of the effect of the respiratory symptoms on the health status measured by SF-36v2 is required.

Based on recent data, approximately two-thirds of the Mongolian population live in cities such as Ulaanbaatar, and the population density in urban areas has been increasing dramatically. Smog covering Ulaanbaatar occurs frequently in winter due to coal or biomass burning, leading to concerns about the health effects of air pollution, especially for children and elderly people who belong to the high-risk group. Recently, Jadambaa et al. listed the environmental risk factors for Mongolia in their systematic review of 59 reports including indoor or outdoor air pollution, metals, environmental tobacco smoke, and other toxic chemicals (Jadambaa et al. 2015). These risk factors are associated with cardiovascular and respiratory diseases in adults and neurodevelopmental disorder in children. However, the health effects of air pollution on respiratory health in Mongolian adults have not yet been fully investigated. Additionally, air quality monitoring and a longitudinal study would be necessary to clarify the association between health status and air pollution in Mongolia.

In addition to air pollution, cigarette smoking including second-hand smoke has been reported to be a cause of lung function decline (Xu et al. 1992; Anthoniesen et al. 2002; James et al. 2005). In the present study, there were no significant differences in health status measured by SF-36v2 between never-smokers and ever-smokers (ex- and current-smokers) in any of the ventilatory impairment groups (Additional file 3: Table S3). Therefore, in the present study, smoking was not suggested as a cause of health status decline but as a cause of lung function decline.

A major limitation in this study is the sampling of participants. The male-to-female ratio was approximately 1:2 because this study was carried out during working hours on weekdays. The sex imbalance may have been contributed by recruitment of passers-by and the sex-difference inherent in the Mongolian lifestyle and culture. Additionally, there were few established local communities in Ulaanbaatar due to the strong tradition of individualism in Mongolians, leading to a lukewarm response to the call for the survey. The female population of the Mongolian population aged 40–79 years was similar (52.7 %) to the male population during 2012–2013 (United Nations 2014), suggesting a potential for selection bias in this study sampling. However, the focus of this study was for validation of the Mongolian SF-36v2. In general, the national norm of the SF-36v2 scores are calculated based on scores obtained from the healthy population aged 20–70 years (Fukuhara and Suzukamo 2004). The survey carried out in this study was intended for people aged 40–79 years, and participants included patients with respiratory impairment who were susceptible to environmental factors such as air pollution. Here, we have demonstrated that the Mongolian SF-36v2 was valid and reliable for both healthy people and in those with respiratory impairment. Further studies in an unbiased population of healthy people representing the Mongolian population is needed to determine the national norm of SF-36v2 scores and the COOP/WONCA charts.

## Conclusions

The results of our present study demonstrated the substantial reliability and validity of the Mongolian SF-36v2, and suggest that the Mongolian SF-36v2 was useful to evaluate the health status of Mongolian adults with normal or abnormal lung function. Health status measured by SF-36v2 was significantly aggravated by combined ventilatory impairment when compared with normal lung function.

## Additional files

10.1186/s40064-016-2204-7 Japanese subjects who visited the healthcare center in the Kumamoto prefecture (Japan) were recruited into our study. Administration of the self-completed questionnaire, body measurements, and medical examination were performed in male and female participants aged 40–79 years, and 855 subjects were eligible for analysis.
10.1186/s40064-016-2204-7 In the Japanese SF-36v2, the RP, GH, BP, and RE subscales consisted of the same items as the English version. Although the VT and MH factors were composed of two factors, the questions making up these subscales were not identical to the English SF-36v2. One of the factors of the VT/MH subscales included questions in the SF subscale. Two of the questions that constituted the PF subscale were classified as another independent factor. The highest factor loadings in each observed variable were expressed as italic.
10.1186/s40064-016-2204-7 There were no significant differences in health status measured by SF-36v2 between never-smokers and ever-smokers (ex- and current-smokers) in any of the ventilatory impairment groups.

## Abbreviations

SF-36v2: Short-form 36 Health Survey version 2
PF: physical functioning
RP: role limitations due to physical problems
BP: bodily pain
GH: general health perceptions
VT: vitality
SF: social functioning
RE: role limitation due to emotional problems
MH: mental health
SpO_2_: peripheral capillary oxygen saturation
FVC: forced vital capacity
FEV_1_: forced expiratory volume in one second
BMI: body mass index
ICC: intraclass correlation coefficient
CI: confidence interval
