# Supplementary material for: Validation of the Mongolian version of the SF-36v2 questionnaire for health status assessment of Mongolian adults
Source: Springerplus. 2016 May 12;5:607. doi: 10.1186/s40064-016-2204-7 (PMC4864778; doi:10.1186/s40064-016-2204-7)
Supplement: Supplementary file 1 — 10.1186/s40064-016-2204-7 Japanese subjects who visited the healthcare center in the Kumamoto prefecture (Japan) were recruited into our study. Administration of the self-completed questionnaire, body measurements, and medical examination were performed in male and female participants aged 40–79 years, and 855 subjects were eligible for analysis. [file 40064_2016_2204_MOESM1_ESM.docx]

Table S1. Demographic characteristics of Japanese subjects

|  | Japanese (n =855) | | |
| --- | --- | --- | --- |
|  | Male | Female | Welch’s *t*-test |
| [n (%)] | 488 (57) | 362 (43) |  |
| Age [mean ± SD] | 57.4 ± 8.0 | 57.8 ± 7.8 | n.s. |
|  |  |  |  |
| Age group [n (%)] |  |  | χ^2^-test |
| 40 – 49 | 69 (14) | 46 (13) | n.s. |
| 50 – 59 | 238 (48) | 177 (49) |  |
| 60 – 69 | 144 (29) | 104 (29) |  |
| 70 – 79 | 41 (8) | 36 (10) |  |
|  |  |  |  |
| Smoking status [(n (%)] |  |  |  |
| Smoker | 127 (26) | 20 (6) | < 0.0001 |
| Ex-smoker | 199 (40) | 16 (4) |  |
| Non-smoker | 166 (34) | 327 (90) |  |
|  |  |  |  |
| Occupation [n (%)] |  |  |  |
| White collar job | 255 (53) | 83 (23) | < 0.0001 |
| Blue collar job | 169 (34) | 150 (41) |  |
| Not in employment | 68 (14) | 130 (36) |  |
| Missing | 0 (0) | 0 (0) |  |
