# Supplementary material for: Validation of the Mongolian version of the SF-36v2 questionnaire for health status assessment of Mongolian adults
Source: Springerplus. 2016 May 12;5:607. doi: 10.1186/s40064-016-2204-7 (PMC4864778; doi:10.1186/s40064-016-2204-7)
Supplement: Supplementary file 2 — 10.1186/s40064-016-2204-7 In the Japanese SF-36v2, the RP, GH, BP, and RE subscales consisted of the same items as the English version. Although the VT and MH factors were composed of two factors, the questions making up these subscales were not identical to the English SF-36v2. One of the factors of the VT/MH subscales included questions in the SF subscale. Two of the questions that constituted the PF subscale were classified as another independent factor. The highest factor loadings in each observed variable were expressed as italic. [file 40064_2016_2204_MOESM2_ESM.docx]

Table S2. Factor analysis of Japanese SF-36v2

| Japanese  n = 855 | | Factor  1 | Factor  2 | Factor  3 | Factor  4 | Factor  5 | Factor  6 | Factor  7 | Factor  8 |
| --- | --- | --- | --- | --- | --- | --- | --- | --- | --- |
| Q1 |  | 0.113 | 0.291 | 0.280 | 0.263 | **0.435** | 0.008 | 0.216 | 0.041 |
| Q2 |  | -0.100 | -0.131 | -0.257 | -0.212 | -0.233 | 0.059 | -0.158 | -0.071 |
| Q3 | (a) | 0.098 | **0.406** | 0.024 | 0.124 | 0.200 | -0.029 | 0.054 | 0.013 |
|  | (b) | 0.120 | **0.608** | 0.077 | 0.058 | 0.138 | 0.134 | 0.064 | 0.121 |
|  | (c) | 0.120 | **0.627** | 0.104 | 0.057 | 0.089 | 0.143 | 0.112 | 0.063 |
|  | (d) | 0.120 | **0.719** | 0.067 | 0.101 | 0.069 | 0.044 | 0.023 | -0.043 |
|  | (e) | 0.083 | **0.651** | 0.090 | -0.002 | 0.041 | 0.243 | -0.017 | -0.029 |
|  | (f) | 0.112 | **0.527** | 0.040 | 0.069 | 0.096 | 0.046 | 0.138 | 0.049 |
|  | (g) | 0.079 | **0.607** | 0.070 | 0.049 | 0.075 | 0.227 | 0.082 | -0.002 |
|  | (h) | 0.059 | 0.534 | -0.005 | 0.015 | -0.003 | **0.638** | 0.016 | -0.011 |
|  | (i) | 0.058 | 0.390 | 0.004 | -0.015 | -0.004 | **0.859** | 0.003 | 0.000 |
|  | (j) | 0.164 | **0.208** | 0.073 | 0.044 | 0.017 | 0.400 | 0.049 | 0.111 |
| Q4 | (a) | **0.722** | 0.151 | 0.152 | 0.050 | 0.132 | 0.077 | 0.078 | 0.117 |
|  | (b) | **0.839** | 0.171 | 0.207 | 0.113 | 0.122 | 0.055 | 0.110 | 0.121 |
|  | (c) | **0.839** | 0.244 | 0.141 | 0.102 | 0.122 | 0.080 | 0.103 | 0.054 |
|  | (d) | **0.817** | 0.199 | 0.197 | 0.094 | 0.105 | 0.077 | 0.103 | 0.106 |
| Q5 | (a) | 0.528 | 0.085 | 0.312 | 0.115 | 0.105 | 0.114 | 0.032 | **0.595** |
|  | (b) | 0.531 | 0.052 | 0.358 | 0.147 | 0.107 | 0.079 | 0.082 | **0.688** |
|  | (c) | 0.488 | 0.075 | 0.410 | 0.169 | 0.101 | 0.054 | 0.055 | **0.592** |
| Q6 |  | 0.230 | 0.127 | **0.405** | 0.185 | 0.149 | 0.036 | 0.075 | 0.154 |
| Q7 |  | 0.132 | 0.204 | 0.190 | 0.124 | 0.169 | 0.048 | **0.845** | 0.021 |
| Q8 |  | 0.282 | 0.268 | 0.200 | 0.118 | 0.223 | 0.032 | **0.642** | 0.076 |
| Q9 | (a) | 0.129 | 0.126 | 0.226 | **0.655** | 0.249 | 0.010 | 0.091 | 0.089 |
|  | (b) | 0.102 | 0.019 | **0.621** | 0.163 | 0.151 | 0.034 | 0.065 | 0.091 |
|  | (c) | 0.185 | 0.059 | **0.779** | 0.145 | 0.100 | 0.014 | 0.036 | 0.123 |
|  | (d) | 0.077 | 0.066 | 0.318 | **0.625** | 0.146 | 0.047 | 0.065 | 0.060 |
|  | (e) | 0.072 | 0.126 | 0.176 | **0.800** | 0.225 | 0.016 | 0.050 | 0.061 |
|  | (f) | 0.141 | 0.081 | **0.782** | 0.223 | 0.117 | -0.033 | 0.034 | 0.101 |
|  | (g) | 0.126 | 0.110 | **0.449** | 0.225 | 0.153 | 0.080 | 0.150 | 0.002 |
|  | (h) | 0.083 | 0.080 | 0.262 | **0.718** | 0.155 | -0.009 | 0.036 | 0.029 |
|  | (i) | 0.123 | 0.040 | **0.423** | 0.293 | 0.219 | 0.058 | 0.191 | -0.004 |
| Q10 |  | 0.276 | 0.227 | **0.388** | 0.100 | 0.149 | 0.031 | 0.068 | 0.136 |
| Q11 | (a) | 0.099 | 0.086 | 0.197 | 0.074 | **0.656** | 0.032 | 0.098 | 0.061 |
|  | (b) | 0.115 | 0.179 | 0.086 | 0.193 | **0.733** | 0.025 | 0.003 | 0.037 |
|  | (c) | 0.079 | 0.111 | 0.216 | 0.139 | **0.580** | -0.029 | 0.123 | 0.045 |
|  | (d) | 0.136 | 0.161 | 0.121 | 0.331 | **0.722** | 0.024 | 0.073 | 0.004 |
