# Supplementary material for: Validation of the Mongolian version of the SF-36v2 questionnaire for health status assessment of Mongolian adults
Source: Springerplus. 2016 May 12;5:607. doi: 10.1186/s40064-016-2204-7 (PMC4864778; doi:10.1186/s40064-016-2204-7)
Supplement: Supplementary file 3 — 10.1186/s40064-016-2204-7 There were no significant differences in health status measured by SF-36v2 between never-smokers and ever-smokers (ex- and current-smokers) in any of the ventilatory impairment groups. [file 40064_2016_2204_MOESM3_ESM.docx]

Table S3. Effect of cigarette smoking on SF-36v2 in each group classified by lung function.

| Lung function | Normal | | Obstructive | | Restrictive | | Combined | |
| --- | --- | --- | --- | --- | --- | --- | --- | --- |
| Smoking experience | Never | Ever | Never | Ever | Never | Ever | Never | Ever |
| SF-36v2 subscales | n = 355 | n = 174 | n = 34 | n = 26 | n = 67 | n = 23 | n = 13 | n = 13 |
| Physical functioning | 64.4 ± 24.7 | 64.9 ± 26.6 | 64.0 ± 20.3 | 63.8 ± 29.4 | 59.7 ± 24.3 | 58.7 ± 27.3 | 47.7 ± 26.3 | 43.1 ± 32.6 |
| Role limitations due to physical health problems | 66.3 ± 25.2 | 66.5 ± 26.2 | 69.9 ± 22.7 | 66.1 ± 28.1 | 64.1 ± 26.7 | 56.3 ± 30.0 | 53.4 ± 27.2 | 42.8 ± 33.5 |
| Role limitations due to emotional problems | 68.5 ± 24.5 | 68.7 ± 26.2 | 73.5 ± 24.3 | 66.3 ± 31.0 | 64.7 ± 27.8 | 59.4 ± 25.2 | 62.2 ± 31.3 | 48.1 ± 38.8 |
| Bodily pain | 59.6 ± 23.9 | 63.0 ± 26.7 | 62.0 ± 25.3 | 64.8 ± 26.2 | 55.0 ± 26.0 | 48.3 ± 25.4 | 35.5 ± 14.9 | 51.8 ± 32.6 |
| General health perceptions | 51.9 ± 22.3 | 52.8 ± 22.1 | 49.3 ± 22.4 | 55.0 ± 19.3 | 49.8 ± 20.8 | 42.3 ± 23.4 | 40.8 ± 23.1 | 39.3 ± 30.4 |
| Vitality | 62.5 ± 18.7 | 59.0 ± 18.4* | 64.2 ± 17.4 | 59.4 ± 22.6 | 60.9 ± 20.9 | 54.3 ± 20.9 | 55.3 ± 18.0 | 47.6 ± 21.3 |
| Social functioning | 73.9 ± 21.8 | 78.2 ± 23.0* | 79.8 ± 21.1 | 76.0 ± 22.3 | 72.2 ± 25.9 | 67.9 ± 24.4 | 68.3 ± 23.2 | 64.4 ± 27.9 |
| Mental health | 69.3 ± 18.4 | 67.9 ± 20.0 | 74.9 ± 16.4 | 65.6 ± 25.2 | 70.7 ± 20.6 | 66.3 ± 22.0 | 65.0 ± 21.2 | 70.4 ± 18.4 |

Data are presented as mean ± SD. ^*^: P < 0.05 vs. Never smoker.
